# Kinetic lattice Monte-Carlo simulations on the ordering kinetics of free and supported FePt L1_0_-nanoparticles

**DOI:** 10.3762/bjnano.2.5

**Published:** 2011-01-17

**Authors:** Michael Müller, Karsten Albe

**Affiliations:** 1 Institut für Materialwissenschaft, Technische Universität Darmstadt, Petersenstr. 32, D-64287 Darmstadt, Germany

**Keywords:** FePt, Monte-Carlo simulations, nanoparticles, ordering kinetics

## Abstract

The ordering kinetics in free and supported L1_0_ nanoparticles was studied by means of lattice-based kinetic Monte-Carlo simulations. Starting from a fully disordered particle of Wulff shape, the simulations show that the nucleation of ordered domains is starting quickly on various (100) facets but is retarded in the particle volume due to the lack of vacancies compared with a thin film geometry. If a substrate is present, we do not find significant differences in the ordering behavior. This holds true, even if we impose a massively increased thermodynamic driving force for interface segregation, because the nucleation of ordered domains on free facets is significantly faster than the bulk diffusion of the segregating species to the interface. In cases where wetting of the substrate or surface facetting occurs, we find that diffusional atomic motion on the surface goes along with an enhanced long-range order.

## Introduction

Nanoparticles in ordered L1_0_ structures like FePt and CoPt are considered as candidate materials for magnetic storage media [1] and biomedical applications [2] because the superparamagnetic limit – where a thermally stable magnetization direction can be expected – is in the range of a 5–10 nm. It has been shown experimentally that ordered arrays of particles with monodisperse size distribution can be prepared by various synthesis routes ([3] and references therein). The as-prepared particles, however, are in most cases partially disordered and can also contain twin planes [4]. In the past, major attention has been paid to the problem of installing internal order without affecting the particle arrangement and size by annealing procedures. As the phase stability of finite phases can be massively affected by the presence of surfaces or interfaces, it was initially even unclear if the single-crystalline ordered configuration represented the thermodynamic equilibrium. At this point, computer simulation studies revealed that nanoparticles larger than 5–6 nm in diameter are single-crystalline and ordered at ambient conditions [5–9], while partially ordered or twinned configurations can be considered as metastable. If the particle composition is properly adjusted (taking into account the surface segregation effects), the ordering temperature is only a weak function of particle size [10]. The question to what extent the ordering kinetics is affected by the small particle size and how the ordering kinetics can be enhanced, has gained much less attention in the past. Rellinghaus et al. [11] concluded from an analysis of particles prepared and annealed in the gas phase, that the ordering kinetics controlled by volume diffusion is relatively slow while Wiedwald et al. [12] showed that the annealing temperature of FePt nanoparticles can be reduced after He-irradiation which points to the fact that athermal vacancies assist in the particle ordering. In principle, ordering requires the rearrangement of atoms on the available lattice sites. Since the L1_0_ structure is close-packed, we can safely ignore that other mechanisms like interstitial diffusion play a role for atomic rearrangements. It is rather the vacancy-mediated site exchange that is responsible for the ordering. Therefore, the ordering kinetics depends on the concentration and mobility of vacancies in the nanoparticle. In a recent paper, we have shown that the thermal vacancy concentration in the inner part of a metallic nanoparticle is significantly reduced [13]. This can be attributed to the excess energy of surface formation that leads to an 1/*r*-dependence of the vacancy formation energy. Considering this, one can anticipate that the ordering kinetics is slower than in the bulk counterpart because of the reduced vacancy concentration. On the other hand, the formation of L1_0_ domains is a nucleation process and therefore the presence of surface sites can facilitate the formation of ordered domains. In addition, the interface to the substrate could also affect the ordering. If we imagine that a (100)-oriented substrate is ideally commensurate with the FePt lattice and one constituent would preferentially segregate to the interface, the L1_0_ domain formation could be promoted in this scenario by thermodynamic driving forces imposing the correct stacking sequence.

In this paper, we present lattice-based kinetic Monte-Carlo simulations of FePt nanoparticles that reveal the influence of free surfaces, bulk vacancies and interaction with a substrate on the disorder–order transition. After describing the methodology, the case of a free particle is studied and compared to a thin film geometry. Then, we investigate the role of a commensurate substrate by varying the thermodynamic driving force for interface segregation. Finally, we conclude with a summary of our results.

## Results and Discussion

### Simulation method and modified Ising-type Hamiltonian

The ordering kinetics in FePt nanoparticles is investigated by an *n*-fold way kinetic Monte-Carlo algorithm [14–15]. The simulations are initialized with a random distribution of Fe and Pt atoms on a fcc lattice. In each step of the simulation, all possible jumps of atoms to neighboring empty lattice sites are identified. To allow a dynamical interpretation of the simulations, the transition rate ν_j_ is calculated for each jump *j* by

[1]
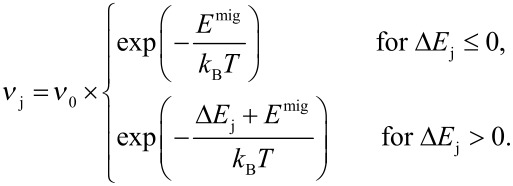


Here, Δ*E*_j_ is the change of energy in the system associated with transition *j* which is calculated using a modification of the Ising-type Hamiltonian with second nearest neighbor interactions as described in [10]. For simplicity, the attempt frequency ν_0_ and the migration barrier *E*^mig^ are assumed independent of the type of the jumping atom or its chemical surrounding. One of the transitions is accepted in each step and a time variable is incremented by Δ*t* = −ln *r* / (∑_j_ ν_j_), where *r* is a random number between 0 and 1.

This kinetic Monte-Carlo algorithm allows to study ordering processes in FePt bulk materials as well as nanoparticles. If a single vacancy is introduced in a lattice with periodic boundary conditions, a rearrangement of atoms by the vacancy diffusion mechanism in bulk materials is simulated. Instead, by initializing the simulation with a particle in the center of an otherwise empty lattice, surface diffusion occurs naturally. As described in [6], the random generation and annihilation of vacancies at the surface then gives rise to a thermal concentration of vacancies in the volume of the particle. In consequence, the absolute values of the interaction energies in the Ising-type Hamiltonian have to be adjusted in order to reproduce reasonable values for the vacancy formation energy. In doing so, important properties like the phase diagram and the segregation behavior of the model described in [10] remain unchanged. The set of modified parameters and the resulting vacancy formation energies 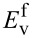 in various chemical surroundings are listed in Table 1. Using these values, the vacancy formation energies in the pure fcc Fe and fcc Pt phases agree well with the experimental values of 1.71 eV for Fe [16] and 1.35 eV for Pt [17], respectively.

**Table 1:** Modified parameters of the Ising-type Hamiltonian of [9] and the resulting vacancy formation energies 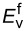 for various configurations.

Parameter	Value [eV]	Vacancy configuration	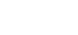

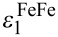	−0.269	vacancy in fcc Fe	1.61
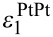	−0.219	vacancy in fcc Pt	1.31
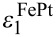	−0.337	Fe site in L1_0_ FePt	1.89
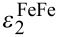	0	Pt site in L1_0_ FePt	1.79
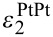	0	Fe site in A1 FePt	1.85
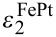	0.00186	Pt site in A1 FePt	1.70

Due to a lack of reference values for the FePt alloy system, experimental data on Pt self-diffusion are used to determine the parameters ν_0_ and *E*^mig^. Ehrhart [17] reports an attempt frequency of ν_0_ = 4 × 10^13^ s^−1^ and an activation energy of 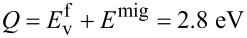 for Pt self-diffusion. As predicted by our Ising-type Hamiltonian, the average formation energy of a vacancy in FePt alloys is 1.8 eV. Therefore, an attempt frequency ν_0_ = 4 × 10^13^ s^−1^ and a migration energy *E*^mig^ = 1 eV should provide realistic estimates for the present simulations.

### Kinetics of ordering in bulk FePt alloys

In order to validate the choice of our model parameters, the ordering kinetics of a FePt bulk crystal predicted by the simulations have been compared to annealing experiments on sputtered FePt thin films at a temperature of 973 K [18]. The bulk crystal has been modeled by periodic boundary conditions applied to a lattice of 70 × 70 × 70 fcc unit cells which corresponds to *N* = 1,372,000 lattice sites. By randomly distributing Fe and Pt atoms and introducing a single vacancy, the lattice has been initialized and the *n*-fold way algorithm described above was used for simulating the dynamics of ordering. In the simulation of a bulk material, the vacancy concentration is fixed at 
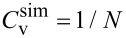
 while in a real system, a temperature dependent vacancy concentration of 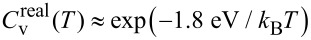 would be observed. In the latter case, the average vacancy formation energy in FePt alloys predicted by the Ising-type Hamiltonian is assumed. In order to re­late the time variable of the *n*-fold way algorithm to a real-time, it has to be scaled by the factor 
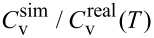
.

**Figure 1:**
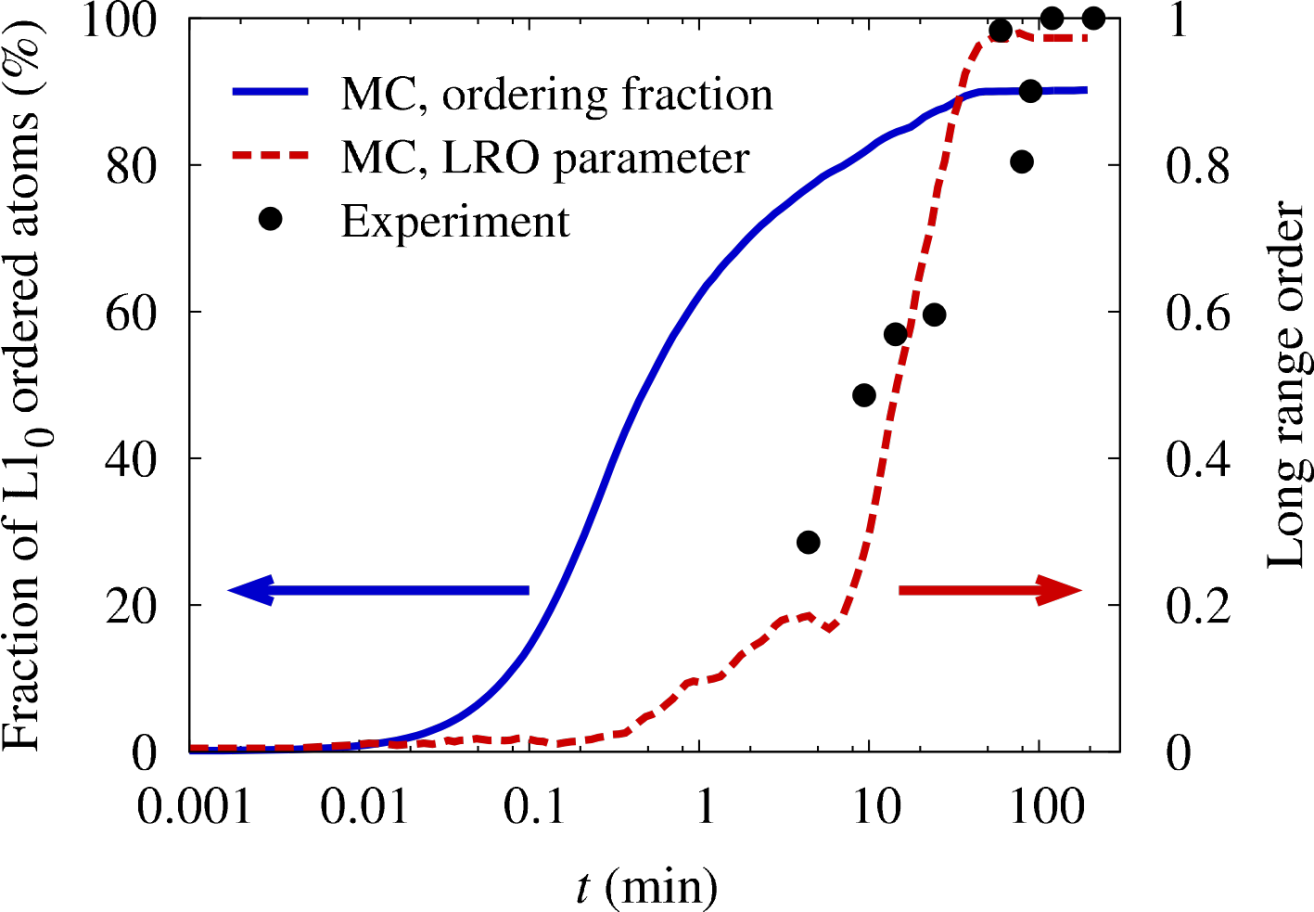
Comparison of simulated ordering kinetics in bulk FePt alloys with results from annealing experiments on sputtered thin films [18]. The temperature in both, experiment and simulation, is 973 K.

At each step of the simulation, the amount of order has been analyzed by calculating the long range order (LRO) parameter as defined in [10] and by counting the fraction of atoms that possess a completely ordered nearest neighborhood. The time evolution of both order parameters is plotted in Figure 1 in comparison to experiments on sputtered FePt films of 10 nm thickness [18]. Because of the large number of atoms, the simulations did not result in a single-domain ordered crystal. Instead, ordered domains with different *c*-axis orientation have been observed. To provide a reasonable measurement of the amount of order, the LRO parameter plotted in Figure 1 has been calculated by summing up the individual domains. A value close to 1, i.e., complete ordering, is obtained after approximately one hour of real-time. Compared to that, the parameter describing the fraction of L1_0_-ordered atoms initially rises on a much shorter time scale, only reflecting the presence of local order. At the end of the simulation the value is well below 1.0 which indicates the fraction of atoms that residing in antiphase boundaries.

In experiments, the presence of different domains has also been observed by dark-field (DF) transmission electron microscopy [18]. In consequence, the total ordered volume fraction has been determined by combining DF images from three independent directions. Therefore, the degree of order reported in [18] corresponds to the additive LRO parameter plotted in Figure 1 and an excellent agreement between the simulations and the experiments can be observed. Given the simplicity of the model and the uncertainties in the parameters ν_0_, *E*^mig^ as well as the vacancy formation energy, such an exact agreement can be considered coincidental to some extent. However, it shows that the chosen values for ν_0_ and *E*^mig^ at least represent a reasonable estimate for diffusion parameters in FePt bulk alloys.

### Free FePt nanoparticles

**Figure 2:**
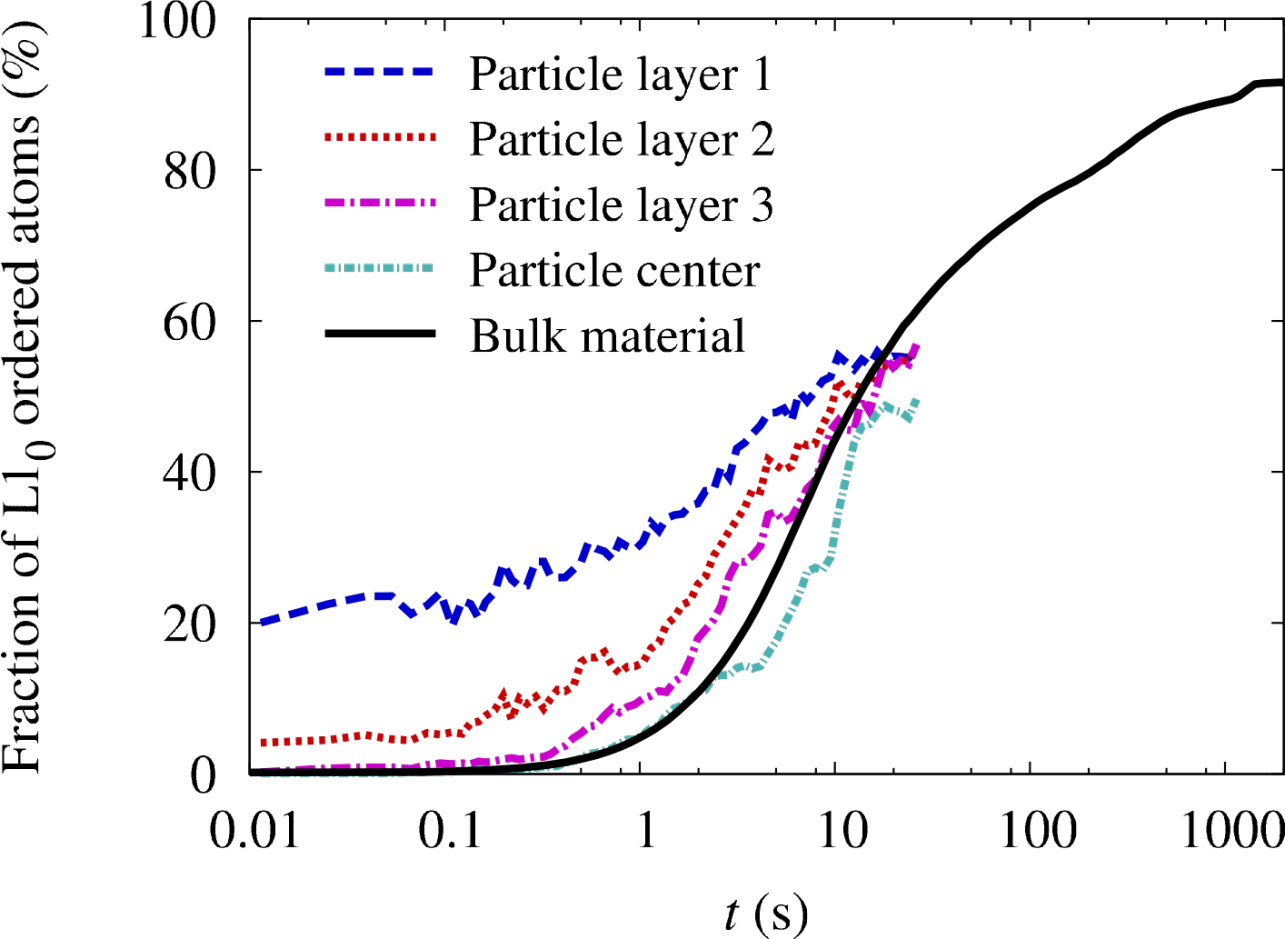
Evolution of ordering fraction in a free FePt nanoparticle of *D* = 5 nm compared to the case of bulk material. For analyzing the contribution of surface and volume effects, the particle has been divided into a spherical core of diameter of 3.2 nm, followed by three layers of thickness of 0.3 nm, with layer 1 denoting the surface. The temperature is 1000 K. Initially, the fraction of ordering was zero in all layers.

By using the model parameters described before, the evolution of ordering was studied for an initially disordered 5 nm particle with closed shells. In this case, nucleation, migration and annihilation of vacancies is fully described by the computer model and no additional assumptions are imposed. In Figure 2, the evolution of the ordering fraction at 1000 K is plotted in comparison to a bulk sample. Since it is expected that surface diffusion can increase the transformation rate in near-surface layers, the particle has been divided into a spherical core of diameter of 3.2 nm, followed by three layers of thickness of 0.3 nm each. By separately analyzing the ordering fraction in all layers, the transition rate can be separated into surface and volume contributions. The effect of surface diffusion is clearly visible in Figure 2. A given amount of ordering is reached within a shorter time scale the closer a layer is positioned to the surface. Compared to the simulations of the bulk crystal, it becomes clear that the ordering in near-surface layers proceeds at a higher rate than in bulk material. In contrast to that, the core of the particle possesses the lowest transformation rate from the disordered to the ordered phase which is due to the lack of vacancies.

**Figure 3:**
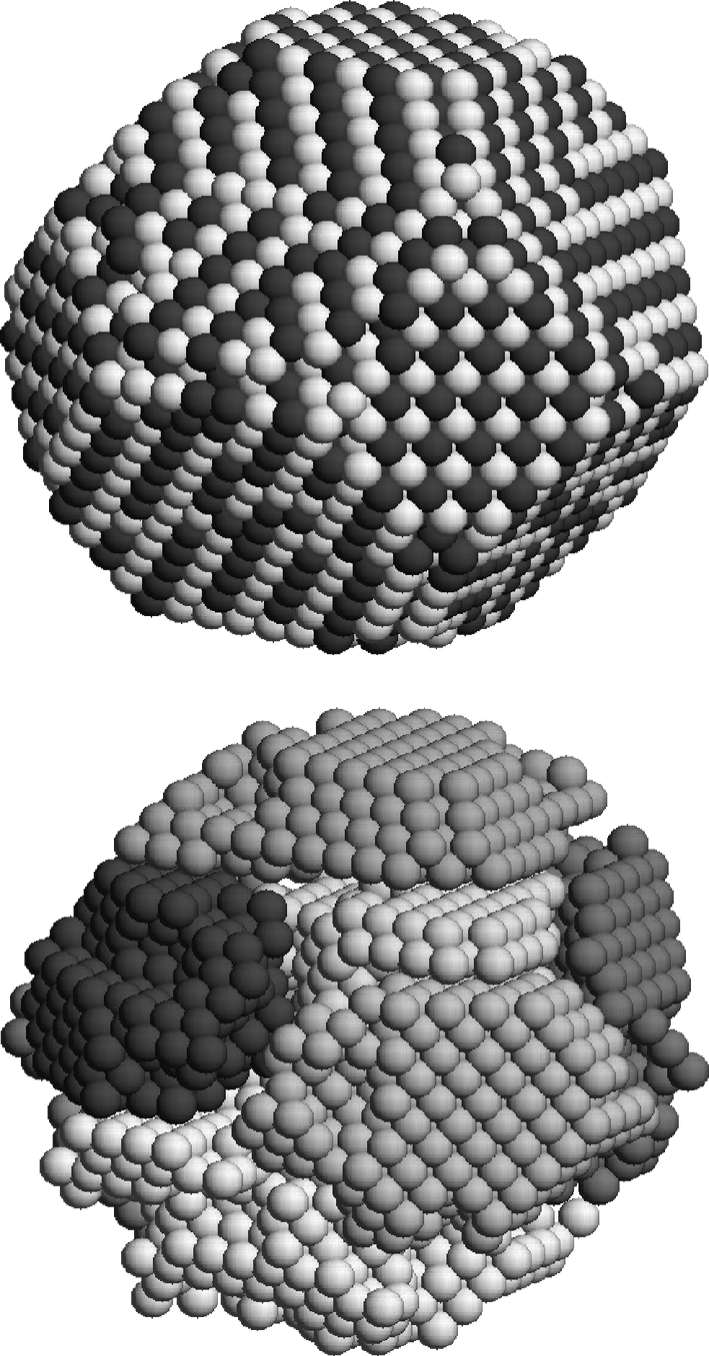
Structure of a free 5 nm particle after 30 s of annealing time at 1000 K. Top: Pt atoms are displayed light gray, Fe atoms dark gray. Bottom: Atoms in antiphase boundaries have been removed and the gray scale visualizes atoms that belong to the same ordered domain.

The kinetic Monte-Carlo simulations of the free FePt nanoparticles were terminated after approximately 30 s of real annealing time which corresponds to one month of computing time. This low efficiency of the algorithm originates from the sampling of the thermal vacancy concentration in the particle. Within the simulated time scale, the ordering fraction plotted in Figure 2 indicates that only 50% of the particle has been transformed. On the other hand, the structure of the particle at the end of the simulation run, which is analyzed in Figure 3, suggests that a rather high degree of order has been achieved. Because of the statistical nucleation of the L1_0_ phase, however, the whole particle is divided into several ordered domains with different *c*-axis orientation. Approximately 50% of the atoms reside in antiphase boundaries, explaining the low value of the ordering fraction.

Due to the existence of multiple domains, the ordering fraction in the bulk material has also reached a value of only 60% after 30 s of annealing time. As can be observed in Figure 2, the annealing process has to be continued for up to 30 min in real-time to eliminate most of the antiphase boundaries. Reaching these time scales in the particle simulations, however, would require computing times of up to 5 years which is not feasible. Even if the ordering process in free FePt nanoparticles could not be followed up to the final stage, options for increasing the transformation rate can be extracted. The effectiveness of these options will be tested in the following. On the one hand, the important contribution of surface diffusion to atomic mobility has been demonstrated. As a consequence, it should be possible to enhance the transformation by increasing the amount of surface diffusion. Furthermore, a random nucleation of L1_0_-ordered domains with different *c*-axis orientation occurs in free particles and long annealing times are needed for obtaining a single domain structure. In order to reduce the random nucleation, a preferential *c*-axis orientation can be induced. By controlling the interface energetics between the particle and a substrate, the possibility for realizing enhanced surface diffusion and a preferential ordering direction in supported FePt nanoparticles is investigated in the following.

### Supported FePt nanoparticles

In order to model the energetics of supported FePt nanoparticles, the lattice Hamiltonian was extended by nearest neighbor bond energies of Fe and Pt atoms to a substrate, denoted by *ε*^FeSub^ and *ε*^PtSub^, respectively. The relative strength of the atom interactions with the substrate was measured by the ratios 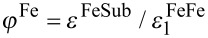 and 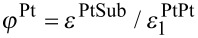. The lattice Hamiltonian restricts the investigations to an epitaxial relation between the substrate and the nanoparticles. Furthermore, only the (100) surface of the substrate was considered.

By varying the strength of the interactions with the substrate, the interface energetics of the supported particles can be modified. Two general cases are considered: First, the relative strength of the interactions of the substrate is assumed to be identical for both elements. In this case, the parameters 
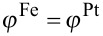
 determine the wetting angle of the particle in equilibrium with the substrate. Second, it is assumed that only Pt has a significant binding energy to the substrate, with 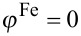 and 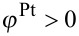. In addition to wetting, the parameters then provide a driving force for segregation of Pt at the interface to the substrate which prefers a *c*-axis orientation of the L1_0_ structure perpendicular to the substrate.

**Figure 4:**
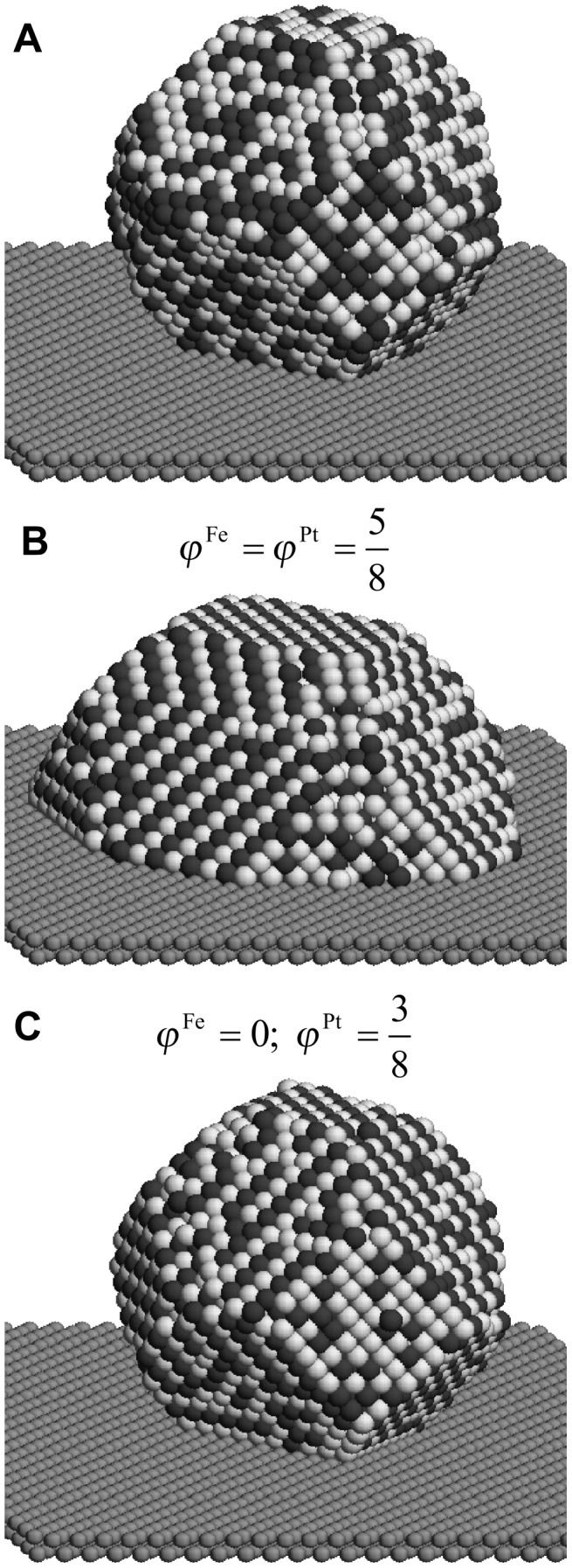
Snapshots of supported FePt nanoparticles illustrating the different interface energetics investigated by the Monte-Carlo simulations. A) A disordered particle of Wulff shape with *D* = 5 nm used at input structure in all simulations. B) Outcome of a simulation with identical Fe-substrate and Pt-substrate interactions leading to strong wetting. C) Outcome of a simulation with only Pt-substrate interactions. The temperature is 1000 K.

For generating the initial configuration for kinetic Monte-Carlo simulations of supported particles, a particle of 5 nm in diameter has been equilibrated at 2 × 10^4^ K. Since melting is excluded in the lattice model, a completely disordered particle of Wulff shape is produced, which is positioned on top of the substrate, as illustrated in Figure 4A.

#### Identical Fe–substrate and Pt–substrate interactions

**Figure 5:**
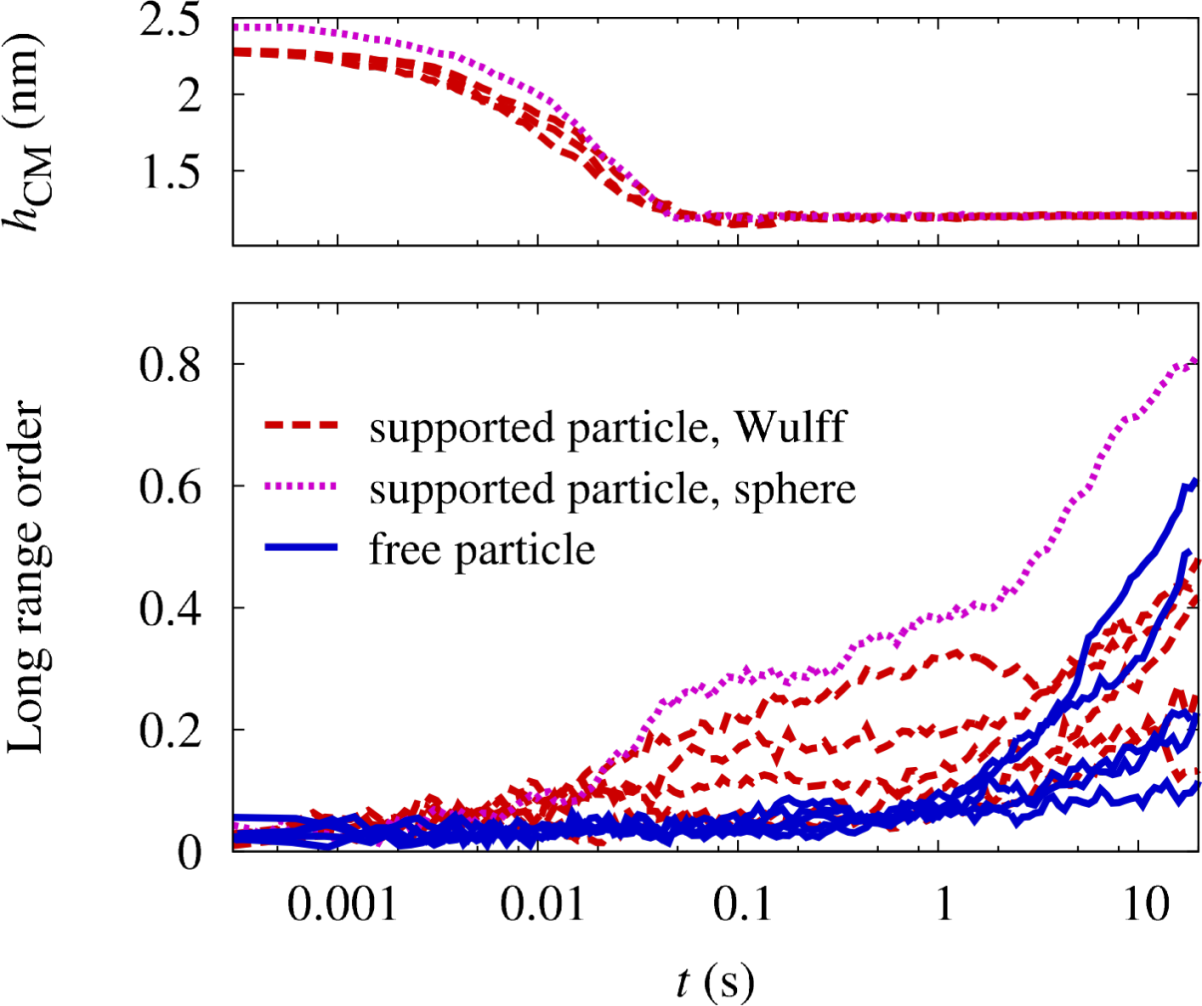
Bottom panel: Evolution of LRO parameter with annealing time at 1000 K in free and supported FePt nanoparticles of *D* = 5 nm and identical Fe–substrate and Pt–substrate interactions. Top panel: Position of the center of mass of supported FePt nanoparticles, measured in height over the substrate *h*_CM_.

If strong wetting of the particle with the substrate occurs, the wetting process after deposition of the particle is accompanied by a considerable amount of surface diffusion. To test if this effect can be used for improving the ordering kinetics in FePt nanoparticles, a strong interaction of Fe and Pt atoms with the substrate (
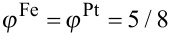
) is assumed. Starting with five different initial configurations for both supported and unsupported particles, annealing simulations were run for 20 s at 1000 K. A typical outcome of a simulation is depicted in Figure 4B, where the strong wetting and the presence of ordered domains is clearly visible. In Figure 5, the evolution of the LRO parameter is analyzed quantitatively and the ordering kinetics is compared to the free particles. The degree of order varies strongly within both classes of particles. This is explained by the presence of multiple ordered domains because a preferred ordering direction is not present. In the supported particles, however, the LRO parameter tends to show a rapid increase up to a value of 0.2 within a short annealing time of less than 0.1 s. During this time, all free particles remain completely disordered. By monitoring the height of the center of mass of the supported particles over the substrate (top panel in Figure 5), this initial increase in ordering can clearly be related to the wetting process. In addition to wetting facetting also contributes to an enhanced ordering if an initially spherical particle is deposited. This can be seen by comparing the time evolution of the long-range order of the spherical with an initially facetted particle.

The mass transport from the particle surface towards the substrate is therefore accompanied by a simultaneous adjustment of order. However, after the wetting process has been completed, the LRO parameter does not increase up to an annealing time of 1 s, from where on the ordering process in the free particles also sets in. Interestingly, the initial increase of ordering in the supported particles does not lead to a higher degree of ordering compared to the free particles at the end of the simulations. The highest LRO parameter is even found in an unsupported particle. The wetting process only takes place during the initial stages of ordering and the enhancement of diffusion in the supported particles does not persist during the coarsening stage at which the number of ordered domains is reduced. Moreover, surface diffusion does not assist in driving out the antiphase boundaries in the particle volume. As the simulations show, strong wetting does not noticeably increase the ordering kinetics.

#### Pt–substrate interactions only

To induce an energetically preferred ordering direction in the particles, only Pt–substrate interactions with 
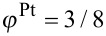
 and 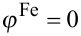 are assumed. As described before, it is not possible to simulate the complete ordering process in FePt nanoparticles within reasonable computing times. Therefore, to compare the ordering in supported particles with a segregation tendency to free particles, the kinetics of the process have been enhanced by artificially increasing the vacancy generation rate. In the simulations of free FePt particles, an average thermal vacancy generation rate of 640 vacancies per second has been measured. By introducing an additional vacancy generation event – which consists of removing a random atom from the interior of the particle with a frequency of 6400 s^−1^ and inserting it on the surface – a tenfold increase of the vacancy generation rate has been achieved in the simulations of this section.

**Figure 6:**
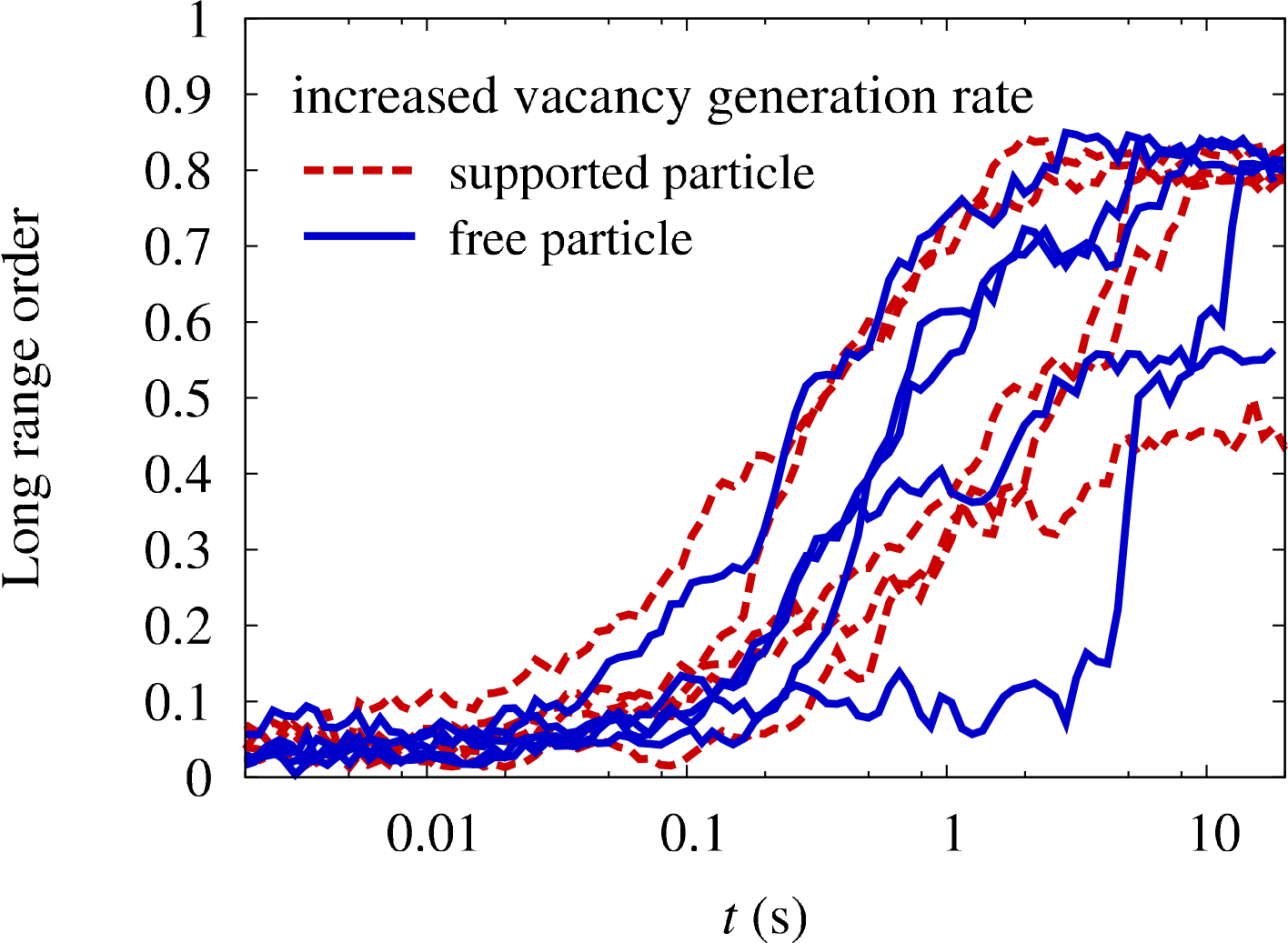
Evolution of LRO parameter with annealing time at 1000 K in free and supported FePt nanoparticles with only Pt-substrate interactions. In order to speed up the simulations, the vacancy generation rate has been increased by a factor ten compared to the thermal vacancy generation rate.

In Figure 4C, the outcome of simulations with an increased vacancy generation rate after an annealing time of 20 s at 1000 K is depicted for a particle with only Pt–substrate interactions. The particle has a truncated octahedral shape and Pt clearly segregates from the interface to the substrate. This segregation imposes a preferential ordering direction with the *c*-axis perpendicular to the substrate, since ordering in other directions generates an antiphase boundary with the segregated layer. However, by comparing the evolution of the LRO parameter in supported and free particles in Figure 6, a positive effect of the segregation on the ordering kinetics cannot be identified. An inspection of the process shows that the segregation of Pt from the interface occurs at a slower time scale than the initial nucleation of ordered domains. Therefore, the energetic preference of the perpendicular direction is not effective during the nucleation stage and the whole ordering process is again limited by the elimination of the antiphase boundaries. Furthermore, the segregation layer only penalizes the wrong ordering directions by the antiphase boundary and has no effect on the volume of the particle. This only leads to small energy differences between perpendicular and horizontal ordering. In result, the prevailing ordering direction at the end of the ordering process is dictated more by statistics than by the global energy minimum.

## Conclusion

The ordering kinetics of FePt nanoparticles has been investigated by kinetic Monte-Carlo simulations based on a modification of the Ising-type lattice Hamiltonian used before [10]. In order to validate the choice of attempt frequencies and migration barriers for atomic diffusion, the variation of ordering with annealing time in a FePt bulk alloy was compared to annealing experiments on sputtered thin films.

From our simulations of free nanoparticles the important influence of surface diffusion on the ordering transition can be seen. In near-surface layers, the A1–L1_0_ transformation proceeds at a higher rate than in the particle core. Because of the statistical nucleation of the ordered phase, however, no single domain particles are obtained and an elimination of the antiphase boundaries could not be observed within the time scales accessible by the simulations. Simulations including a crystalline support show that, even if strong interface segregation is assumed, no influence on the disorder–order transition can be expected. The fact that free, non-supported facets have easier access to free volume for generating vacancies, leads to a higher nucleation rate for ordered domains on free facets compared to the migration rate of segregating species. In conclusion, our study reveals the physical reasons for the slow installment of order in L1_0_ nanoparticles: Firstly, the relative lack of bulk vacancies and secondly, the occurrence of antiphase boundaries which are due to the nucleation of ordered domains on various (100) facets. Our results suggest that generating athermal vacancies by simultaneous irradiation with ions during the annealing procedure could be a means for improving internal order in L1_0_ nanoparticles.
